# Carnitine-related hypoglycemia caused by 3 days of pivalate antibiotic therapy in a patient with severe muscular dystrophy: a case report

**DOI:** 10.1186/s12887-017-0835-7

**Published:** 2017-03-14

**Authors:** Masanori Ito, Mitsumasa Fukuda, Yuka Suzuki, Hiroyuki Wakamoto, Eiichi Ishii

**Affiliations:** 1 Department of Pediatrics Ehime University Graduate School of Medicine, 454 Shitsukawa, Toon, Ehime 790-0295 Japan; 2 Ehime Rehabilitation Center for Children, Toon, Ehime Japan

**Keywords:** Carnitine, Fukuyama-type congenital muscular dystrophy, Antibiotics, Pivalic acid, Hypoglycemia

## Abstract

**Background:**

Long-term treatment with antibiotics containing pivalic acid may decrease serum carnitine concentration and can sometimes be associated with severe hypoglycemia and encephalopathy in infants. Little has been reported, however, on severe hypocarnitinemia induced by acute administration in older children.

**Case presentation:**

We describe a 6-year-old Japanese girl with Fukuyama-type congenital muscular dystrophy who lost consciousness after 3 days of treatment with an antibiotic containing pivalic acid (cefditoren pivoxil). Investigations at the onset of unconsciousness revealed hypoglycemia (free plasma glucose concentration: 31 mg/dL) and hypocarnitinemia (serum free carnitine concentration: 6.2 μmol/L). Intravenous administration of glucose rapidly improved her symptoms without any complications. Serum free carnitine concentration was 29.0 μmol/L immediately prior to the initiation of cefditoren pivoxil. Computed tomography scanning showed severe peripheral skeletal muscle atrophy, indicating the likelihood of decreased carnitine stores in skeletal muscle.

**Conclusions:**

Although serum carnitine concentration can appear deceptively normal, skeletal muscle carnitine stores can be reduced in patients with severe muscular atrophy. Even a short course of a pivalate-containing antibiotic can lead to life-threatening hypocarnitinemia in older children with severe muscular dystrophy.

## Background

Carnitine, a water-soluble quaternary amine, is responsible for the intracellular transport of long-chain fatty acids into mitochondria, facilitating fatty acid oxidation. Severe carnitine deficiency impairs β-oxidation, and thereby the ability to produce glucose, which may result in hypoglycemia. Although most patients with carnitine deficiency are asymptomatic, it can cause muscle weakness, hypotonia, nausea and vomiting, fatigue, recurrent infection, failure to thrive, poor appetite, poor concentration, apathy, and headaches. Furthermore, carnitine deficiency can occasionally cause severe and life-threatening complications, including hypoglycemic encephalopathy and dilated cardiomyopathy [1, 2].

Carnitine deficiency may be classified as primary or secondary; the primary form is associated with genetically determined metabolic errors whereas the secondary form is associated with acquired diseases or iatrogenic factors such as drug administration. Recent studies have reported that severe secondary carnitine deficiency induced by long-term administration of pivalate-containing antibiotics, particularly for more than 14 days, causes hypoketotic hypoglycemia and acute encephalopathy in infants [3–6]. However, there have been few reports to date of carnitine deficiency provoked by short-term antibiotics in older children. Here, we report the case of a 6-year-old girl with Fukuyama-type congenital muscular dystrophy (FCMD) who developed severe hypoglycemia caused by carnitine deficiency after a 3-day oral course of cefditoren pivoxil (CDTR-PI), an antibiotic containing pivalic acid.

## Case presentation

A 6-year-old Japanese girl with FCMD was admitted to our hospital with sudden-onset impaired consciousness. She had been diagnosed with FCMD by genetic testing at 9 months of age, having a homo-retrotransposon insertional mutation of the *fukutin* gene. Her height was 108 cm (−1.0 SD) and weight was 13.0 kg (−2.0 SD). She was able to sit but not stand, and could eat only a soft diet. Her Gross Motor Function Classification System score was level five [7]. She had previously been admitted to hospital for treatment of acute pharyngitis, for which she was prescribed CDTR-PI (8.5 mg/kg/day orally). During this time, she could consume half of her usual calorific intake without weight loss or dehydration. After 3 days of treatment, her level of consciousness declined. On physical examination, her Glasgow Coma Scale score was 9 out of 15 (E3V2M4), core temperature was 36.6 °C, blood pressure was 108/68 mmHg, and heart rate was 130 beats/min. Laboratory investigations revealed a serum creatine kinase concentration of 588 IU/L and hypoglycemia (free plasma glucose concentration of 31 mg/dL). Hepatic and renal function, serum electrolyte and ammonia concentrations, and acid-base balance were all within the normal range, but serum free carnitine concentration was markedly reduced (6.2 μmol/L) (Table 1).

**Table 1:** Blood examination, chest X-ray, and echocardiographic findings on admission

Hematological examination						
Complete blood count			Biochemistry		Blood gas (vein)	
WBC	1.8 × 103/μl		TP	7.1 g/dl	pH	7.428
RBC	3.5 × 106/μl		AST	65 U/l	pO2	46.4 mmHg
Hb	12.9 g/dl		ALT	38 U/l,	pCO2	39.9 mmHg
Plt	38.9 × 105/μl		Na	135 mEq/l	HCO3-	26.3 mmol/l
			K	4.2 mEq/l	BE	1.8 mmol/l
			Cl	97 mEq/l	Lac	9.0 mmol/l
			BUN	13 mg/dl		
			Creatinine	<0.10 mg/dl		
			CK	588 IU/l		
			CRP	0.24 mg/dl		
			NH3	52 μg/dl		
			Glucose	31 mg/dl		
Serum carnitine				Serum carnitine (just 3 days before cefditoren pivixil was started)		
Total Carnitine		14.1 μmol/l		Total Carnitine	33.7 μmol/l	
Free Carnitine		6.2 μmol/l		Free Carnitine	29.0 μmol/l	
Acyl Carnitine		4.7 μmol/l		Acyl Carnitine	7.9 μmol/l	
Chest X-ray				Echocardiographic study		
Cardiothoracic ratio		50%		IVC diameter 11 mm		
Lungs		normal		Caval/Ao ratio 0.9 (normal value: 0.8–1.0)		

**Fig. 1:**
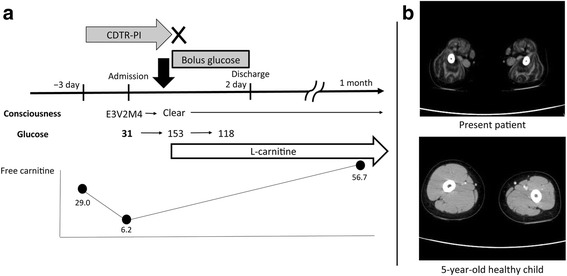
Clinical course and computed tomography (CT) scanning of the central part of the femoral muscles. **a** Clinical course of the patient from the onset of coma 3 days after initiation of treatment with cefditoren pivoxil (CDTR-PI) to follow-up 1 month later. Serum l-carnitine and glucose concentrations are shown. **b** CT scans of the femur acquired to assess skeletal muscle volume, which was markedly less than would be expected in a healthy child

We diagnosed the patient with hypoglycemia originating from a carnitine deficiency induced by CDTR-PI, as the serum free carnitine concentration was 29.0 μmol/L when measured retrospectively in a sample taken 3 days before CDTR-PI treatment was initiated. The patient was immediately administered intravenous glucose and her level of consciousness rapidly improved without any complications. We discontinued CDTR-PI treatment and initiated l-carnitine supplementation. One month later, the patient’s serum free carnitine concentration lay within the normal range with no relapse of symptoms, hypoglycemia, or side effects reported (Fig. 1a).

Computed tomography (CT) scanning undertaken to evaluate skeletal muscle volume showed severe atrophy of the peripheral muscles compared with an age-matched healthy child who had undergone CT to investigate left leg pain (Fig. 1b). We also calculated the renal reabsorption rate of free carnitine (RRFC) using the following equation: RRFC (%) = 1 − (urine free carnitine × serum creatinine)/(serum free carnitine × urine creatinine). An RRFC of 98% (normal value: >95%) indicated that renal carnitine malabsorption was not responsible for the patient’s episode of hypocarnitinemia [8]. Serum creatinine concentration was <0.1 mg/dL in this case; therefore, we substituted 0.1 mg/dL and considered that the true RRFC value must have been above 98%.

## Discussion

Our patient suffered severe carnitine deficiency induced by 3-day oral administration of CDTR-PI. Pivalic acid is released as a result of the metabolism of the prodrug CDTR-PI, and combines with serum free carnitine to form pivaloylcarnitine, which is excreted by the kidneys. Consequently, long-term treatment with an antibiotic containing pivalic acid may provoke hypocarnitinemia [3–6, 9], especially in patients at risk of carnitine deficiency such as those with inherited causes of carnitine metabolism, severe epilepsy, renal disorders or severe neurologic disability, infants less than 2 years old, and patients fed parenterally or taking multiple antiepileptic drugs [10, 11]. However, a case has been reported of a 1-year-old Japanese patient with hypoglycemia associated with hypocarnitinemia induced by 2 days of cefcapene pivoxil treatment [12]. Additionally, Ito et al. reported that treatment with cefteram pivoxil significantly decreased the level of serum free carnitine in both children and adults, even in short-term therapy, and advised that carnitine supplementation may be necessary for patients who are taking these antibiotics, particularly those vulnerable to carnitine deficiency [13]. Although our patient was an older child who could usually receive adequate calories orally (1200 kcal/day, appropriate for her body weight and activity level), she did not receive enough to eat for 3 days (about half of her usual calorific intake) after antibiotic treatment was initiated because of her acute pharyngitis.

We judge, however, that even if our patient’s diet had been marginally deficient in carnitine, her most important risk factor for carnitine deficiency was severe muscular dystrophy. Large quantities of carnitine can be stored in skeletal muscle; in a healthy patient, the proportion stored in skeletal muscle exceeds 95% [1]. Consequently, severe muscle atrophy such as that seen in patients with Duchenne or Becker muscular dystrophy [14, 15] reduces the capacity to store carnitine and replenish serum free carnitine when it is suddenly depleted, even when the renal reabsorption of carnitine is normal. As far as we are aware, there have been no previous reports of carnitine deficiency in FCMD, but our patient’s skeletal muscle volume was markedly lower than that of a healthy child. We suggest that the patient’s reduced muscle bulk would have further predisposed her to hypoglycemia, which was likely to the result of a lack of glycogen and glycogenic amino acid storage in skeletal muscle as well as β-oxidation impairment. Fever would also have induced a hypercatabolic state that would have markedly elevated glucose consumption.

We did not measure blood and urine ketone bodies, a diagnostic limitation for hypoketotic hypoglycemia in this patient, because we imitated prompt emergency treatment for severe hypoglycemia to prevent permanent central nervous system damage. However, the normal venous blood gas test results arguably preclude the possibility of ketotic hypoglycemia. Early diagnosis and rapid, appropriate treatment for severe hypoglycemia resolved our patient’s symptoms without any complications.

The rapid fall in serum free carnitine from 29.0 μmol/L to 6.2 μmol/L observed in this girl with FCMD after just 3 days of treatment with CDTR-PI underlines the speed at which potentially life-threatening symptoms can develop in patients at risk of carnitine deficiency. It is important to note that pivalate antibiotics are not the only type of antibiotic that can influence serum free carnitine; β-lactam antibiotics are reported to competitively block the binding of organic cation/carnitine transporter 2 (OCTN2) and inhibit reabsorption in the kidney [16, 17]. As far as we are aware, however, there have been no reports of β-lactam antibiotics causing adverse events such as those seen in this case.

## Conclusions

This case shows that even short-term administration of antibiotics containing pivalic acid in older children with severe musculoskeletal disorders requires careful monitoring for carnitine deficiency to avoid serious adverse effects. Alternative antibiotics should therefore be administered to children at risk. Supplementation with l-carnitine is recommended so that complications can be avoided if alternative antibiotics cannot be identified.

## Abbreviations

CDTR-PI: Cefditoren pivoxil
CT: Computed tomography
FCMD: Fukuyama-type congenital muscular dystrophy
OCTN2: Organic cation/carnitine transporter 2
RRFC: Renal reabsorption rate of free carnitine
